# Draft Genome Sequence of the Bisphenol A-Degrading Bacterium *Sphingobium* sp. Strain YL23

**DOI:** 10.1128/genomeA.00549-13

**Published:** 2013-08-01

**Authors:** Anyi Hu, Min Lv, Chang-Ping Yu

**Affiliations:** Key Laboratory of Urban Environment and Health, Institute of Urban Environment, Chinese Academy of Sciences, Xiamen, China

## Abstract

*Sphingobium* sp. strain YL23, a novel bacterium isolated from sewage sludge of a domestic wastewater treatment plant, has been shown to completely degrade bisphenol A under aerobic conditions. Here, we describe a 3.8-Mb assembly of its genome sequence and major findings from its annotation.

## GENOME ANNOUNCEMENT

Bisphenol A (BPA), a synthetic chemical widely and abundantly used in the production of polycarbonates and epoxy resins in many consumer products, has aroused particular concerns due to its endocrine-disrupting effects and widespread human exposure (1). Partially removed BPA in treated wastewater also led to its ubiquitous distribution in the environment (2). Although microbial degradation has been shown to be important for BPA removal in the environment (3), little is known about the genetic mechanisms of BPA degradation in microorganisms.

To gain insight into the mechanisms involved in microbe-mediated BPA degradation, we determined the draft genome sequence of a novel BPA-degrading bacterium, *Sphingobium* sp. strain YL23, which was isolated from the sewage sludge of a full-scale domestic wastewater treatment plant in Fujian Province, China. The phylogenetic analysis based on 16S rRNA gene sequences indicated that YL23 was most closely related to two type strains, *Sphingobium chlorophenolicum* L-1^T^ and *Sphingobium japonicum* UT26S^T^, with 97.8% and 97% sequence identities, respectively.

The genome of YL23 was sequenced using the Illumina Solexa GAII instrument with a paired-end library. A total of 532.4 Mb sequences were produced, providing approximately 140-fold coverage. Genome sequences were assembled *in silico* using SOAPdenovo2 (4), resulting in 67 contigs (>300 bp) with an *N*_50_ length of 148,657 bp. The coding sequences (CDS) were predicted by using Glimmer 3.02 (5) and the RAST server (6), while tRNAs and rRNAs were identified by using tRNAscan-SE (7) and RNAmmer (8), respectively. The functions of the CDS were then annotated through comparisons with the databases of NCBI-NR, COG, and KEGG (9).

The YL23 draft genome sequence comprised 3.8 Mb, with an average GC content of 63.7%. A total of 3,795 CDS, 1 16S-23S-5S operon, and 40 tRNAs were predicted in the genome. Comparison with the genome sequences of *S. Chlorophenolicum* strain L-1^T^ and *S. japonicum* strain UT26S^T^, performed by using mGenomeSubtrator (10), demonstrated that 2,571 CDS were conserved among three strains, with 859 strain-specific CDS present in YL23 (BLASTP E value, ≤1e-5; identity, ≥30%; coverage, ≥70%). An average nucleotide identity (ANI) analysis showed that YL23 shared a low degree of similarity with strains L-1^T^ and UT26S^T^ (<81% ANIb and <90% ANIm), suggesting that YL23 may represent a novel species of the genus *Sphingobium* (11).

Batch degradation experiments demonstrated that YL23 degraded 60 mg/liter BPA within 24 h under aerobic conditions, and two BPA degradation products of the cytochrome P450 monooxygenase system (P450s), 1,2-bis(4-hydroxyphenyl)-2-propanol and 2,2-bis(4-hydroxyphenyl)-1-propanol, were detected using liquid chromatography coupled to high-resolution mass spectrometry. In addition, the P450s was found in the YL23 genome and showed high similarity to the previously reported P450s of *Sphingomonas bisphenolicum* AO1 involved in BPA degradation (12). Further in-depth genomic analysis is needed to provide more information for elucidation of the genetic mechanism of BPA degradation, including genome organization and the evolution of the degradation pathway.

### Nucleotide sequence accession numbers. 

This whole-genome shotgun project has been deposited at DDBJ/EMBL/GenBank under the accession number ASTG00000000. The version described in this paper is the first version, ASTG01000000.
